# Monolithically integrated white light LEDs on (11–22) semi-polar GaN templates

**DOI:** 10.1038/s41598-018-37008-5

**Published:** 2019-02-04

**Authors:** N. Poyiatzis, M. Athanasiou, J. Bai, Y. Gong, T. Wang

**Affiliations:** Department of Electronic and Electrical Engineering, University of Sheffield, Mappin Street, Sheffield, S1 3JD United Kingdom

## Abstract

Carrier transport issues in a (11–22) semi-polar GaN based white light emitting diode (consisting of yellow and blue emissions) have been investigated by detailed simulations, demonstrating that the growth order of yellow and blue InGaN quantum wells plays a critically important role in achieving white emission. The growth order needs to be yellow InGaN quantum wells first and then a blue InGaN quantum well after the growth of n-type GaN. The fundamental reason is due to the poor hole concentration distribution across the whole InGaN quantum well region. In order to effectively capture holes in both the yellow InGaN quantum wells and the blue InGaN quantum well, a thin GaN spacer has been introduced prior to the blue InGaN quantum well. The detailed simulations of the band diagram and the hole concentration distribution across the yellow and the blue quantum wells have been conducted, showing that the thin GaN spacer can effectively balance the hole concentration between the yellow and the blue InGaN quantum wells, eventually determining their relative intensity between the yellow and the blue emissions. Based on this simulation, we have demonstrated a monolithically multi-colour LED grown on our high quality semi-polar (11–22) GaN templates.

## Introduction

General illumination is one of the major sources for electricity demand globally. Due to global warming and the impending energy crisis, it is crucially important to develop energy-saving solid-state lighting (SSL). White light-emitting diodes (LEDs), which are primarily based on III-nitride semiconductor LEDs, are expected to ultimately replace incandescent bulbs and fluorescent tubes for a host of outdoor and indoor lighting applications due to the advantages of low power consumption and long lifetime^1–3^. The demand driven by energy saving has made the development of III-nitride based optoelectronics emerge as one of the fastest growing semiconductor areas over the last two decades.

So far, the “blue LED + yellow phosphor” approach is maintaining its strong lead for the fabrication of white LEDs^4,5^. The performance of such white LEDs has almost approached its limit, but is still far from the requirements described in the US road map for developing SSL^6^. Furthermore, the phosphor-converted approach suffers from numerous drawbacks, such as down-conversion losses, optical losses due to backscattering, heat related effects and the degradation of yellow phosphor as a result of its long-term exposure^7–9^. In order to address these great challenges, a number of approaches have been proposed, such as monolithically integrated hybrid III-nitride/colloidal quantum dots^10,11^; hybrid III-nitride/organic conjugated polymers^12–15^. Although these white LEDs are still based on a down-conversion approach, they demonstrate a unique non-radiative energy transfer effect, which cannot be achieved by the “blue LED + phosphor” white LEDs mentioned above.

One of the most direct routes for the fabrication of monolithic white LEDs is to utilize InGaN quantum wells with different emission wavelengths, where these emissions with different wavelengths can be obtained by controlling either InGaN quantum well (QW) thickness or indium content in InGaN^16–20^. In this approach, a combination of either blue/green/red (RGB) emissions or blue/yellow emissions is required. In principle, this approach not only is cost-effective but also matches the current growth and fabrication techniques for III-nitride optoelectronics. However, two major challenges need to be addressed before the potential of this approach can be possibly achieved. The first challenge is to obtain long wavelengths such as green and yellow emission with high performance. Current III-nitride LEDs are grown on *c-plane* substrates. The polar orientation poses strain-induced piezoelectric fields due to the lattice-mismatch between InGaN and GaN, which is the so-called quantum-confined Stark effect (QCSE). As a result, internal quantum efficiency is reduced, and drops significantly further when InGaN quantum wells move towards longer wavelengths such as the green or yellow spectral region (where higher indium content is required, leading to an enhancement in QCSE), thus forming the well-known “Green-Yellow gap” phenomenon. Furthermore, the *c-plane* GaN also leads to fundamental limitations in incorporating indium into GaN^21,22^. The second issue is due to the complicated carrier transport in InGaN QWs with different indium composition as a result of much lower hole mobility and hole concentration than those of electrons, potentially leading to severe non-uniform carrier distribution across all the InGaN QWs involved. This complexity is further enhanced by InGaN structures grown on *c-plane* GaN due to piezo-electrical fields induced polarisation. This issue becomes even more complicated with increasing indium content as a result of an enhancement in piezo-electrical fields induced polarisation. So far, there is no systematic study addressing this issue.

Growth of III-nitrides along a semi-polar direction, in particular the (11–22) orientation, would be a promising solution to achieve long wavelength emissions, as this orientation is expected to lead to not only significantly reduced piezoelectric polarization fields but also enhanced indium incorporation efficiency in InGaN^23,24^. Furthermore, an increasing demand on Li-Fi applications requires a white LED with an ultra-fast response. Current blue LEDs on *c-plane* substrates suffer from a long carrier recombination lifetime as a result of QCSE, typically on a scale of a few to 10 nanoseconds for blue emission and ~100 nanoseconds for green emission^25^. Phosphors generally exhibit even a longer response time, typically on a microsecond scale. In contrast, semi-polar (in particular (11–22)) InGaN quantum wells exhibit a much shorter carrier recombination lifetime, typically hundreds of picoseconds for blue emission^26^. Therefore, semi-polar phosphor-free LEDs are ideal for Li-Fi applications. Recently, Sizov *et al*.^27^ observed severe non-uniform carrier distribution among the InGaN multiple quantum wells (MQWs) of a laser diode grown on a *c-plane* substrate, leading to an increase in threshold current when the number of InGaN MQWs is above 2, while they did not observe this phenomenon on the LDs grown on semi-polar substrates. This fact also indicates that semi-polar orientation facilitates the distribution of injection current across InGaN QWs along the vertical direction.

All the above facts demonstrate that (11–22) semi-polar GaN is potentially an ideal candidate which can meet all the requirements for the fabrication of monolithic white LEDs with multiple-colour emissions for simultaneous general illumination and Li-Fi applications. However, one of the greatest challenges is due to the lack of semi-polar GaN with high crystal quality on industry-compatible substrates, such as sapphire. Recently, our group has demonstrated semi-polar (11–22) InGaN LEDs on our overgrown semi-polar GaN templates with significantly improved crystal quality^28,29^, leading to semi-polar InGaN LEDs with high performance in a wide spectral region of up to amber^30^.

In this paper, by means of performing detailed simulations, carrier transport issues in (11–22) semi-polar white LEDs with two different InGaN quantum wells (blue and yellow) have been studied. This study has been further compared with its *c-plane* counterpart, demonstrating the major advantages of employing semi-polar (11–22) GaN for the growth of monolithic white LEDs. Based on the simulation results, we have designed semi-polar (11–22) LEDs with multiple colour emission for a validation purpose. Two different kinds of LED structures, labelled as Sample A and Sample B, have been designed and then have been grown on our high quality semi-polar overgrown GaN, where in both cases the LEDs consist of an InGaN single quantum well (SQW) for blue emission and two pairs of InGaN MQWs for yellow emission. However, the growth order of the blue SQW and the yellow MQWs is different. For Sample A, the blue SQW is grown first, followed by the yellow MQWs, while for Sample B the yellow MQWs are grown first and then the blue SQW afterwards. In both cases, the growth conditions remained identical.

**Figure 1:**
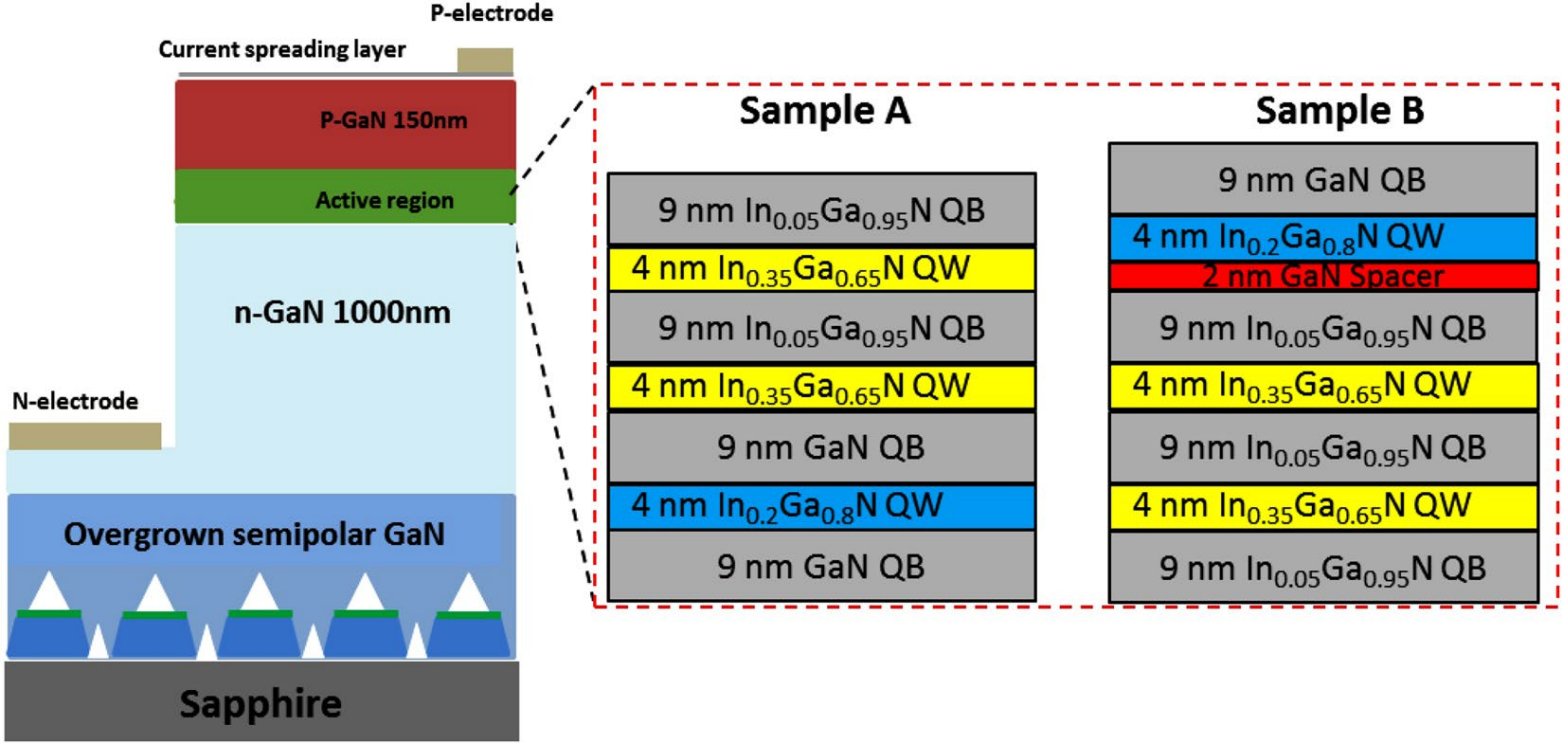
Schematics of the structures of Sample A and Sample B.

Figure 1 illustrates schematically the structures of Sample A and Sample B. Both LEDs consist of a 1 µm n-type GaN layer, then an active region comprising a blue InGaN SQW with low indium content and 2 pairs of yellow InGaN MQWs with high indium content, and finally a 150 nm p-type GaN layer. Figure 1 also provides the detailed parameters including indium composition and the thicknesses of quantum wells and barriers. For Sample B, an extra GaN spacer with a thickness of 2 nm is introduced prior to the growth of the blue SQW, for which we will discuss about later. The structure of Sample B is particularly interesting in the present study, which will be explained later.

Initially, band-diagram simulations have been performed on a dual-colour LED (consisting of blue and yellow) with an identical structure grown on a (11–22) GaN surface and a *c-plane* GaN surface in order to study the effect of the crystal orientation on carrier transportation. In both cases, the LED structure simulated consists of two pairs of InGaN MQWs for yellow emission followed by a blue SQW. The thicknesses of the quantum well and the barrier are 4 nm and 9 nm, respectively. Despite the fact that the indium incorporation is naturally limited in *c-plane* LED, both LEDs are designed to have similar emission wavelength. The donor and acceptor concentrations used for the simulations are 5 × 10^18^ cm^−3^ and 5 × 10^17^ cm^−3^, respectively, which are fairly standard.

**Figure 2:**
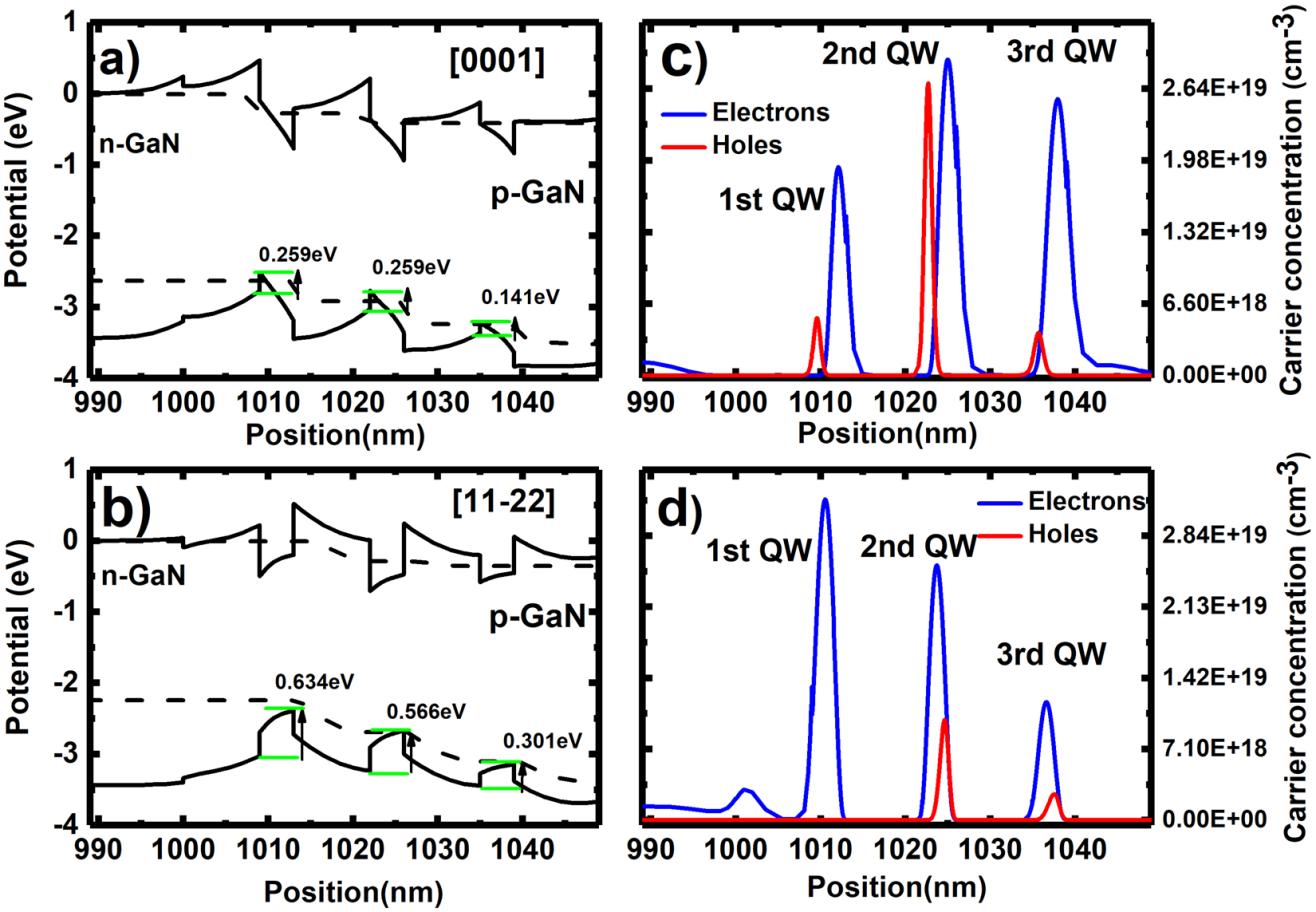
Simulated band diagrams of (**a**) *c-plane* LED and (**b**) (11–22) LED; and their corresponding carrier concentration in the InGaN quantum well region of (**c**) *c-plane* LED and (**d**) (11–22) LED.

Band diagram simulations have been conducted using SiLENSe 5.11 package^31^. Figure 2 shows the simulation results of the two cases. The simulation of band diagrams and the carrier distribution of *c-plane* and semi-polar (11–22) LEDs have been conducted as a function of injection current density of up to 180 A/cm^2^ (equivalent to 200 mA on a standard LED with a size of 330 × 330 µm^2^, while the injection current density for practical applications cannot be beyond 180 A/cm^2^.). Figure 2a,b show their band diagrams at 180 A/cm^2^, while Fig. 2c,d provide their carrier distribution at 180 A/cm^2^. Due to the much higher mobility and lower effective mass of electrons than those of holes, electrons can overcome the potential barrier and can be distributed across the whole InGaN QW region without any great concerns as shown in Fig. 2c,d. Of course, the electron concentration of n-type GaN is generally one or even two orders of magnitude higher than that of p-type GaN. Previous studies have shown that semi-polar LEDs exhibit an opposite polarity in comparison with *c-plane* LEDs^32^. Therefore, the polarization induced electric field in InGaN MQWs leads to an additional energy barrier for holes. As expected the crystal orientation plays a significant role in hole transport and then hole concentration distribution.

Figure 2a,b show an enhanced barrier potential for holes in the semi-polar (11–22) LED in comparison with the *c-plane* LED. In detail, the potential barrier for holes between the 1^st^ grown InGaN QW and the 3^rd^ grown InGaN QW is significantly enhanced compared with the *c-plane* LEDs as a result of reversed polarisation induced electrical field, effectively decelerating holes to inject to the 1^st^ grown QW thus reducing the holes captured in the 1^st^ InGaN QW. Therefore, the hole concentration in the 1^st^ grown QW of the semi-polar LED is much less than that of its *c-plane* counterpart as shown in Fig. 2c,d.

Figure 2c,d also show that the hole concentration in the 3^rd^ grown QW for both cases is comparable, but is much lower than that in the 2^nd^ grown QW. As a consequence, the emission from the 3^rd^ grown QW is expected to be very weak. In this case, if such an LED is grown on either c-plane or (11–22) semi-polar plane, it is highly likely that only the yellow emission can be observed. This case is even worse for the *c-plane* LED as a result of strong QCSE. It is worth highlighting that it is important to grow the 3^rd^ grown QW for blue emission, where the indium content is generally low. If the 3^rd^ QW is for yellow emission (i.e., Sample A), the situation becomes even worse. The fundamental reason is due to very weak confinement for holes in the 3^rd^ grown QW, leading to very low hole concentration in the 3^rd^ grown QW, which has been confirmed by Fig. 2c,d. This will be further discussed later.

In order to take advantage of utilising semi-polar LEDs and also enhance the confinement for the holes in the 3^rd^ grown InGaN quantum well, we are proposing a new structure. A thin GaN spacer is introduced, namely, a thin GaN barrier is grown prior to the blue SQW.

**Figure 3:**
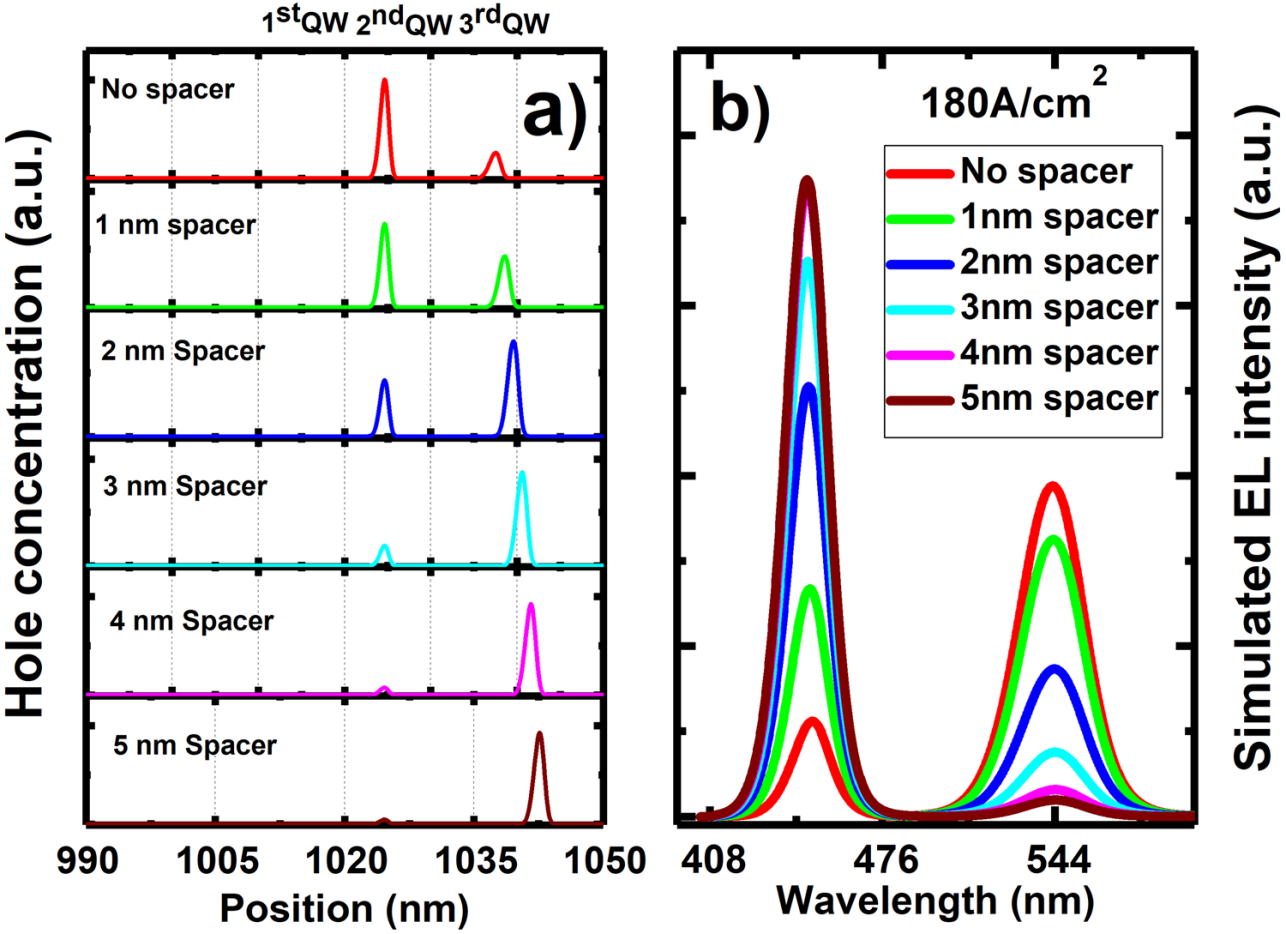
(**a**) Calculated distribution of hole concentration across the InGaN quantum well region as a function of GaN spacer thickness; (**b**) Simulated electro-luminesce (EL) spectra as a function of GaN spacer thickness.

Figure 3a shows the calculated distribution of hole concentration across the 2^nd^ and the 3^rd^ grown QWs. With increasing GaN spacer thickness, the carrier concentration in the 3^rd^ grown QW increases significantly, while the hole concentration in the 2^nd^ grown QW decreases quickly. Considering the overall hole concentration in both the 2^nd^ and 3^rd^ grown QWs, the optimised thickness of the GaN spacer is 2 nm. Figure 3b shows the simulated electro-luminescence (EL) spectra as a function of GaN spacer thickness. In terms of the relative EL intensity of the emission from the 2^nd^ grown QW (yellow) and the 3^rd^ grown QW (blue), the optimised LED is the one with a 2 nm GaN spacer.

Finally, we may conclude that the optimised structure for a dual colour LED can be either Sample A or Sample B. For sample B, a 2 nm GaN spacer is employed in order to enhance the confinement for the 3^rd^ grown InGaN QW as discussed above.

**Figure 4:**
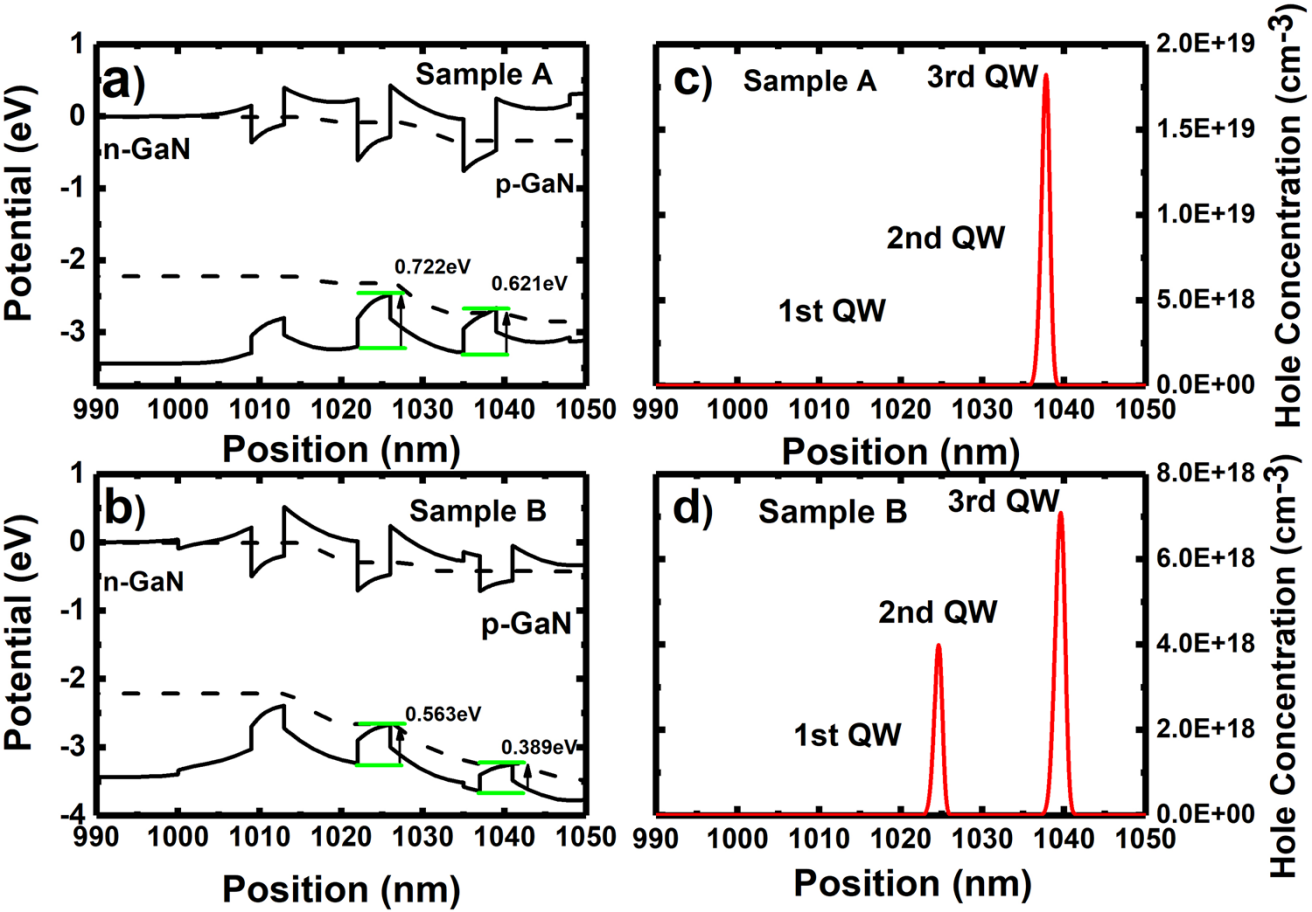
Simulated band diagrams for (**a**) Sample A and (**b**) Sample B, and simulated hole concentration in the InGaN quantum well region of (**c**) Sample A and (**d**) Sample B.

Band diagrams and hole distribution have been performed on Sample A and Sample B as a function of injection current density of up to 180 A/cm^2^. Figure 4a,b show the simulated band diagrams at 180 A/cm^2^, while Fig. 4c,d correspond their simulated hole concentration distribution. If a blue SQW is grown first followed by the yellow InGaN QWs (i.e., Sample A) as shown in Fig. 4a, the 3^rd^ grown QW exhibits an energy difference of 0.621 eV between the quantum well and the barrier for holes due to high indium composition in the yellow QWs. As a result, it is difficult for holes to escape from the 3^rd^ grown InGaN QW and thus remain trapped in the 3^rd^ grown InGaN QW (i.e., yellow emission). Therefore, the hole concentration in the 2^nd^ grown QW is extremely low as shown in Fig. 4c. In contrast, for sample B, where the yellow quantum wells are grown first followed by the blue SQW with a 2 nm GaN spacer as discussed above, the energy barrier for the 3^rd^ grown QW has significantly dropped by 0.232 eV compared with Sample A as a result of the low indium composition in the 3^rd^ grown QW (i.e., for blue). Due to the reduction in energy barrier for holes, it becomes possible for holes to escape from the 3^rd^ grown QW to the 2^nd^ grown QW, and thus the hole concentration in the 2^nd^ grown QW increases substantially as shown in Fig. 4d. Therefore, in this case, dual colour emission can be possibly achieved.

In order to validate the above idea, we have grown Sample A and Sample B on our overgrown semi-polar (11–22) GaN with high crystal quality^28–30^, where regularly arrayed (11–22) GaN micro-rods with a diameter of 4 μm used as a template for overgrowth are fabricated by means of using a standard photolithography mask patterning technique and then dry etching processes. The overgrown semi-polar (11–22) GaN is around 5 μm. Refs^28–30^ provide further detailed procedures for obtaining such high quality semi-polar (11–22) GaN with detailed material characterization. A standard lithography technique and dry-etching processes have been used to fabricate LED chips with a standard size of 330 × 330 µm^2^. A 7 nm/7 nm Ni/Au alloy was deposited by electron beam deposition and then annealed by rapid thermal annealing as transparent p-type contact. N-type contact was formed on n-type GaN by depositing a Ti/Al/Ti/Au alloy. Ti/Au was deposited as a pad electrode on both p-type and n-type contacts. All the measurements were carried out on bare-chip devices at room temperature in a continuous wave (CW) mode, using a LCS-100-UV characterization system equipped with a CCD APRAR spectrometer.

**Figure 5:**
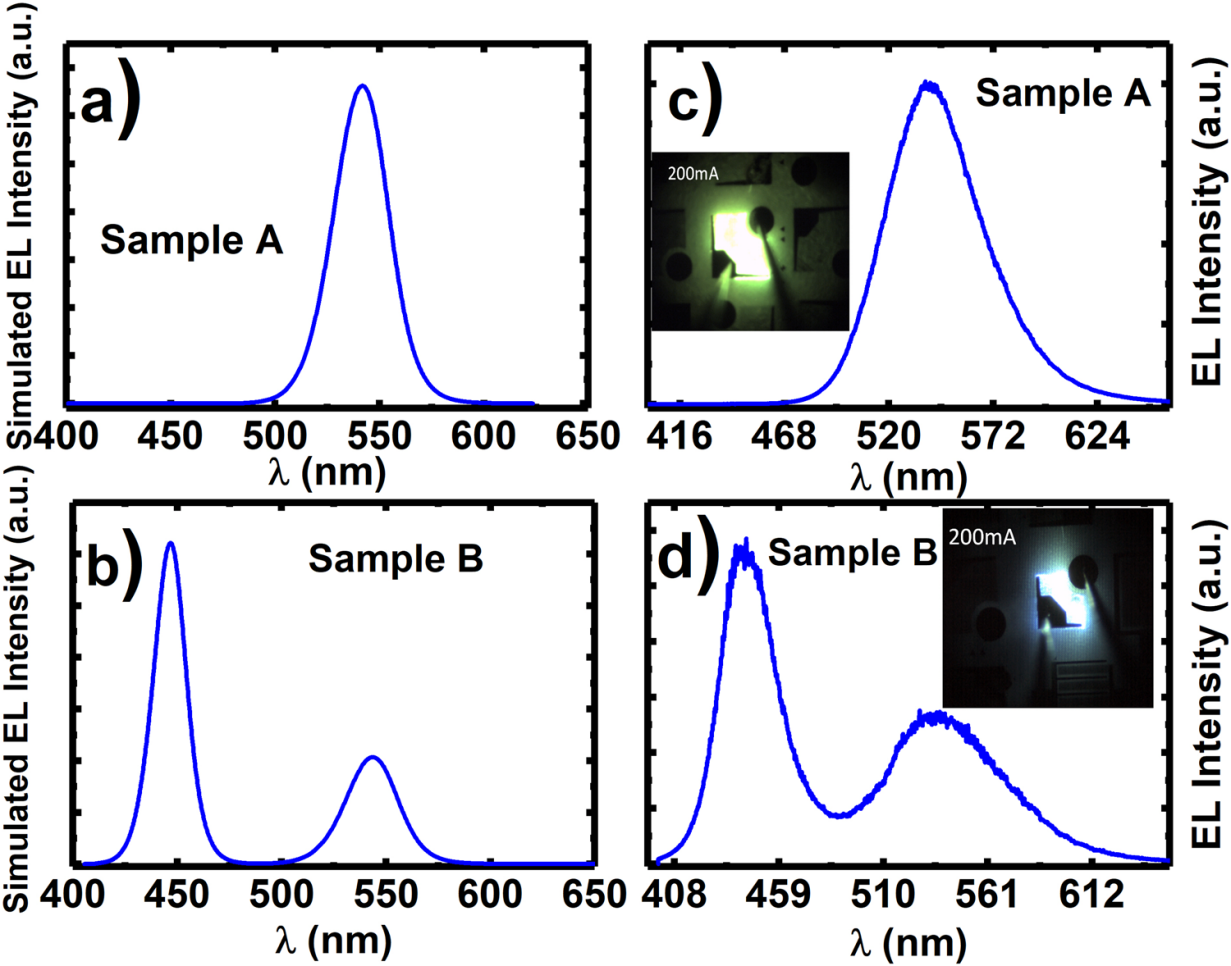
Simulated EL for (**a**) Sample A and (**b**) Sample B at 180 A/cm^2^; and measured EL spectra of (**c**) Sample A and (**d**) Sample B taken at 180 A/cm^2^. The insets show the EL images of Sample A and Sample B taken at 180 A/cm^2^.

Figure 5a,b illustrate the simulated EL spectra of Sample A and Sample B at 180 A/cm^2^, whereas Fig. 5c,d show the experimental EL at the same injection current density, where Sample A shows single yellow emission at 545 nm, while sample B shows dual emission at 450 and 545 nm. The insets of Fig. 5c,d also include EL images of Sample A and Sample B, taken at 180 A/cm^2^. Sample A shows an overall higher integrated intensity with a factor of ~2.4 compared with Sample B. The simulated and experimental EL spectra are in good agreement, supporting our discussion. This means that, in order to achieve dual emission, yellow QWs will have to be grown first followed by a blue SQW. Another important point is that a thin GaN spacer needs to be grown prior to the blue SQW. The results also indicate that the hole transportation issue is vitally important for designing a dual emission LED.

In summary, detailed simulations have been performed in order to investigate carrier transport issues in (11–22) semi-polar LEDs with multiple colours, which consist of yellow InGaN QWs and a blue InGaN QW. The simulation results show that the growth order of these yellow and blue InGaN QWs plays a critically important role in achieving white emission. In order to achieve white emission, yellow InGaN quantum wells have to be grown, followed by a blue InGaN quantum well. Otherwise, there is only single colour emission. In order to capture the holes in both the yellow InGaN quantum wells and the blue InGaN quantum well, a thin GaN spacer is required prior to the blue InGaN SQW in order to effectively balance the carrier concentration between the yellow InGaN quantum wells and the blue InGaN quantum well, which eventually determine their relative intensity between yellow and blue emission. Based on this simulation, we have demonstrated a monolithically multi-colour LED grown on our high quality semi-polar (11–22) GaN templates.

## Methods

### Simulations

SiLENSe 5.11 package has been used to perform the simulations of band diagrams, carrier concentration and electron-luminescence spectra. The simulations are conducted using a one-dimensional drift-diffusion model based on the self-consistent solution of the Poisson equations for the electrostatic potential and the Fermi-Dirac statistics for carrier concentration in active regions. Although semi-polar InGaN LEDs exhibit reduced polarisation induced electrical fields, our simulations have taken into account the polarization effect in addition to radiative recombination and non-radiative recombination as a result of defects (the defect density used for the simulation is taken from our previously study, for which one can refer to ref.^28–30^). The light emission spectra are simulated by solving the self–consistent Poisson and Schrödinger equations for electron and hole wave functions for each quantum well. The complex structure of the valence band of III nitride materials is obtained within the 8 × 8 Kane Hamiltonian. For further detailed information, please refer to ref.^33^. For the material parameters used for our simulation, please refer to Supplementary Information.

### Epitaxial growth

High quality (11–22) semi-polar GaN templates on sapphire are used for the MOVPE growth of multi-colour LED structures, where the dislocation density and the basal stacking fault density of the (11–22) semi-polar GaN templates are typically 2.0 × 10^8^ cm^−2^ and 2.8 × 10^4^ cm^−1^, respectively. After the templates are subject to an initial annealing process under hydrogen ambient at a high temperature, a 400 nm un-doped GaN layer is grown, which is followed by a 1 µm n-type GaN layer, then an active region comprising a blue InGaN SQW with low indium content and 2 pairs of yellow InGaN MQWs with high indium content, and finally a 150 nm p-type GaN layer.

## Supplementary information

Supplementary info
