# Supplementary material for: Monolithically integrated white light LEDs on (11–22) semi-polar GaN templates
Source: Sci Rep. 2019 Feb 4;9:1383. doi: 10.1038/s41598-018-37008-5 (PMC6361879; doi:10.1038/s41598-018-37008-5)
Supplement: Supplementary file 1 — Supplementary info [file 41598_2018_37008_MOESM1_ESM.pdf]

Monolithically integrated white light LEDs on (11-22) semi-polar GaN templates

N. Poyiatzis, M. Athanasiou, J Bai, Y. Gong and T. Wang*

Department of Electronic and Electrical Engineering, University of Sheffield, Mappin Street, Sheffield S1 3JD, United Kingdom

*E-mail: t.wang@sheffield.ac.uk

Supplementary Information

Table 1: Basic GaN materials parameters

Parameter	Units		AlN	GaN	InN
**Lattice constant**	nm	α	0.3111	0.3182	0.3540
**Dielectric constant**		ϵ_{33}	8.5	8.9	15.3
**Piezoelectric tensor**	C/m^2	e_{31}	-0.58	-0.33	-0.22
**Piezoelectric tensor**	C/m^2	e_{33}	1.55	0.65	0.43
**Elastic Constant**	GP_a	C_{13}	115	105	95
**Elastic constant**	GP_a	C_{33}	385	395	200
**Spontaneous Polarization**	C/m^2	P^{sp}	-0.081	-0.029	-0.032
**Band gap**	eV	E_g	6.2	3.4	0.7

Table 2: Effective electron and hole masses in binary nitrides at 300K.

	**AlN**		**GaN**		**InN**	
	⊥	‖	⊥	‖	⊥	‖
m_n	0.25	0.25	0.2	0.2	0.1	0.1
m_{lh}	1.95	0.25	1.1	0.15	1.35	0.1
m_{hh}	1.95	2.58	1.1	1.65	1.35	1.45
m_{so}	0.23	1.93	0.15	1.1	0.09	1.54

The ionization energies are E_D=13meV and $E_A = 170meV$ for donors and acceptors, respectively.
